# Naringenin prevents non‐alcoholic steatohepatitis by modulating the host metabolome and intestinal microbiome in MCD diet‐fed mice

**DOI:** 10.1002/fsn3.3700

**Published:** 2023-09-27

**Authors:** Peng Cao, Ming Yue, Yuanlei Cheng, Mitchell A. Sullivan, Wen Chen, Huifan Yu, Fei Li, Sanlan Wu, Yongning Lv, Xuejia Zhai, Yu Zhang

**Affiliations:** ^1^ Department of Pharmacy, Union Hospital, Tongji Medical College Huazhong University of Science and Technology Wuhan China; ^2^ Hubei Province Clinical Research Center for Precision Medicine for Critical Illness Wuhan China; ^3^ Hubei Key Laboratory of Wudang Local Chinese Medicine Research, School of Pharmaceutical Sciences Hubei University of Medicine Shiyan China; ^4^ Hubei Key Laboratory of Biological Targeted Therapy, Union Hospital, Tongji Medical College Huazhong University of Science and Technology Wuhan China; ^5^ Department of Pharmacy, The Central Hospital of Wuhan, Tongji Medical College Huazhong University of Science and Technology Wuhan China; ^6^ Glycation and Diabetes, Mater Research Institute – The University of Queensland Translational Research Institute Brisbane Queensland Australia

**Keywords:** gut microbiota, metabolomics, multi‐omics, naringenin, NASH

## Abstract

Non‐alcoholic steatohepatitis (NASH) is a severe inflammatory phase of the non‐alcoholic fatty liver disease (NAFLD) spectrum and can progress to advanced stages of NAFLD if left untreated. This study uses multi‐omics data to elucidate the underlying mechanism of naringenin's reported benefit in alleviating (NASH). Male mice were fed a NASH‐inducing (methionine‐choline‐deficient) MCD diet with or without naringenin supplementation for 6 weeks. Naringenin prevented NASH‐induced histopathological liver damage and reversed the abnormal levels of hepatic triglyceride (TG)/total cholesterol (TC), serum TG/TC, serum alanine aminotransferase/aspartate transaminase, and hepatic malondialdehyde and glutathione. Importantly, naringenin intervention significantly modulated the relative abundance of gut microbiota and the host metabolomic profile. We detected more than 700 metabolites in the serum and found that the gut genus levels of *Anaeroplasma* and the *[Eubacterium] nodatum group* were closely associated with xanthine, 2‐picoline, and securinine, respectively. *Tuzzerella* alterations showed the highest number of associations with host endogenous metabolites such as FAHFA (8:0/10:0), FFA (20:2), carnitine C8:1, tridecanedioic acid, securinine, acetylvaline, DL‐O‐tyrosine, and Phe‐Asn. This study indicates that the interplay between host serum metabolites and gut microbiota may contribute to the therapeutic effect of naringenin against NASH.

## Graphical abstract

This study provides mechanistic insights into how naringenin suppresses NASH development. Naringenin intervention substantially restored the serum metabolome and gut microbiome in a NASH setting. Through the integration of multi‐omics data and correlation analysis, we found that the genera levels of *Anaeroplasma*, *[Eubacterium]_nodatum*_group, and *Tuzzerella* were significantly altered by naringenin treatment and closely correlated with 17 kinds of host serum endogenous metabolites, such as fatty acyl and amino acid derivatives.

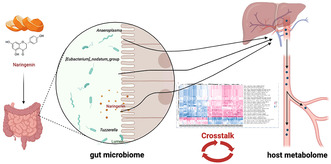

## 1 INTRODUCTION

Nonalcoholic fatty liver disease (NAFLD) is a common, multifactorial, and complicated liver disease with an increasing incidence worldwide (Friedman et al., 2018). The development of NAFLD is primarily attributed to unhealthy lifestyles and is manifested by the pathological accumulation of lipid droplets within hepatocytes. Nonalcoholic steatohepatitis (NASH) is a severe condition on the NAFLD spectrum that affects nearly 400 million people worldwide and can progress to liver fibrosis, cirrhosis, and liver cancer, posing significant health risks to humans. NASH cannot be reversed with lifestyle adjustments alone; therapeutic drug interventions are necessary. To date, there have been numerous clinical trials aimed at treating NASH; however, current therapeutic options remain limited and inadequate to prevent disease progression. Therefore, it is imperative to develop new strategies to combat this disease.

The use of natural products in the development of drugs often benefits from low toxicity and high activity; thus, naturally existing compounds have broad and promising applications in the development of new therapies (Molaei et al., 2021; Rahaman et al., 2023; M. Zhang et al., 2023). Flavonoids are an important class of compounds with potent antioxidant and anti‐inflammatory capacities (Cao, Gan, et al., 2023; Cao, Wang, et al., 2023). Naringenin, a flavonoid abundant in citrus fruits, has been demonstrated to reduce lipid accumulation in the liver, making it promising in the treatment of NAFLD (Sui et al., 2018). Accumulating literature reports that naringenin exerts a significant hepatoprotective effect (Hernandez‐Aquino & Muriel, 2018). Previous studies have shown that the therapeutic benefits of naringenin for NAFLD may be related to the regulation of gut bacterial composition (Mu et al., 2020). Another study demonstrated that naringenin alleviated NASH by activating SIRT1‐mediated signaling cascades in the liver (Hua et al., 2021).

Phenolic antioxidants such as quercetin, catechin, epigallocatechin gallate, and kaempferol have been widely studied in the context of treating NAFLD. Quercetin, which is the most reported polyphenol, has been demonstrated to ameliorate NAFLD in mice via regulating gut microbiota (*Akkermansia*, *Bifidobacterium*, and *Lactobacillus*) and altering the host fatty acid metabolism (Shi et al., 2022). Catechin can alleviate NASH by altering the host hepatic metabolome (Sasaki et al., 2021). Epigallocatechin gallate, also an antioxidant polyphenol, prevents NASH by affecting the gut microbiota (Dey et al., 2020). Kaempferol has been shown to improve NASH via a mechanism of regulating the host serum metabolome (Lu et al., 2020). However, an in‐depth understanding of the beneficial mechanism of phenolic antioxidants from the perspective of multi‐omics such as gut microbiome and metabolomics has not been well studied.

The balance between gut microbiome and host metabolome has become recognized as important in the maintenance of physiological function and has become a target in treating various diseases (Cao, Hu, et al., 2023; Guo et al., 2022; Zhang et al., 2022). Currently, the integrated analysis of metabolomics and 16S ribosomal RNA (rRNA) microbial gene sequencing is a common approach to studying the underlying mechanisms of drug action or disease pathology (Yang et al., 2019). The sequencing of 16S rRNA bacterial genes is an accurate and fast method for microbial classification and is widely employed to measure the composition and richness of the microbiota. Metabolomics uses complex analytical techniques to comprehensively identify changes in an organism's metabolic spectrum. Feces and serum are commonly used to analyze the composition of the gut microbiome and the host metabolic profile, respectively. Profiling of both may reveal potential mechanisms of interaction between the gut microbiome and the host.

Gut bacteria influence host lipid metabolism, and an imbalance in the microbiome has been shown to be a novel mechanism involved in NAFLD (Huang et al., 2021). Dysregulation of the gut microbiota is involved in various metabolic diseases such as NAFLD, diabetes, and obesity. Changes in gut microbiota composition can independently lead to obesity, which is the most important risk factor for NAFLD. It remains unclear the extent to which gut bacteria affect the host's metabolic profile and whether modulating gut–host interactions might be a new strategy for the treatment of NAFLD. Up to now, both metabolomic and microbiome signatures have been studied separately in NASH, as recently reviewed (Masoodi et al., 2021); however, the crosstalk between them has not been uncovered. To our knowledge, only one study has reported the protective mechanism of naringenin on NAFLD in terms of gut bacterial dysbiosis (Mu et al., 2020); however, they did not analyze the interactions between the microbiome and host, making it ambiguous to elucidate the specific role of gut bacteria in this process. Therefore, we aimed to uncover potential mechanisms by integrating metabolomics with microbiome analysis.

Metabolomics, a relatively novel big data analytic approach after the genomic and proteomic eras, can directly reflect the response of an organism to a pathological condition or to a therapeutic approach. This is particularly useful in studying the phenotype of complex metabolic diseases like NASH (Cao et al., 2021, 2022; Cao, Gan, et al., 2023; Li et al., 2022). Metabolomics can be divided into two types: untargeted and targeted. Currently, non‐targeted metabolomics is frequently used to find differential metabolites in biological matrices, as it can produce a full scan of metabolic profiles. By using an untargeted metabolomic approach, Masarone et al. found altered metabolic profiles among various stages of the NAFLD spectrum. They used this data on endogenous metabolites to construct mathematical models that could distinguish and predict each clinical stage with an accuracy of over 80%. However, the accuracy of this method is limited as it identifies unknown compounds without the benefit of including standard references. Thus, misidentification is common in non‐targeted metabolomics, and targeted metabolomics that employ known standards are often required to further validate the chosen metabolite of interest. The protective effects of naringenin have been demonstrated to be associated with restoring metabolic profiles in various liver diseases, including CCl_4_‐induced and acetaminophen‐induced liver injury (Ammar et al., 2022; Lin et al., 2022). The influence of naringenin on the metabolic profile of preclinical models of NASH has not been investigated until this present study.

Herein, we established a diet‐induced NASH mouse model to evaluate the hepatoprotective effect of naringenin. We then conducted a multi‐omics study using serum metabolomics and 16S rRNA gene sequencing (gut microbiome analysis) to comprehensively explore the potential mechanism of naringin in the prevention of NASH. Our findings provide novel insight into the pharmacological activity of naringenin, and this information may help facilitate the development of bioactive natural products for the treatment of NASH.

## 2 METHODS

### 2.1 Experimental materials and animals

Naringenin was obtained from Aladdin Bio‐Chem Technology Co., Ltd. The methionine‐choline‐deficient (MCD) diet, customized MCD diet containing naringenin (0.05%, w/w), and isocaloric AIN93G diet were produced at Medicence Lab Animal Diets Co., Ltd. The naringenin‐containing MCD diet is approximately equivalent to a dosage of 80 mg/kg/day, which has been shown to exert its pharmacological effects (Khodayar et al., 2020).

SPF‐grade C57BL/6J mice (male, 6‐week‐old, 18–22 g) were purchased from Three Gorges University (SYXK 2022–0012) and kept in an SPF‐grade room (12‐h light and dark cycle, 25°C). The animal study was reviewed and approved by the Institutional Animal Care and Use Committee of Tongji Medical College, Huazhong University of Science and Technology. After 1 week of acclimatization, they were randomly divided into 3 groups (*n* = 8 per group) and fed for 6 weeks: (1) Normal group: mice fed with an isocaloric AIN93G diet; (2) NASH group: mice fed with an MCD diet; (3) Naringenin group: mice fed with a customized MCD diet containing naringenin. At the end of the animal experiment, the blood, colon contents, and liver tissues of each mouse were collected.

Biochemical indicators, including triglyceride (TG), total cholesterol (TC), alanine aminotransferase (ALT), aspartate transaminase (AST), malondialdehyde (MDA), and glutathione (GSH), were measured according to the specifications of commercial kits (Nanjing Jiancheng Bioengineering Institute). All other experimental materials were commercially available.

### 2.2 Profiling of the serum metabolome and gut microbiome

For the serum metabolome, an instrument of ultra‐performance liquid chromatography (UPLC, ExionLC AD) and tandem MS (triple quadrupole‐linear ion trap MS (QTRAP®)) was used, and two separate LC conditions, including T3 and Amide modes, were employed in the analysis. The LC and MS conditions were consistent with our previous publications (Cao et al., 2019, 2021; Guo et al., 2022). Based on the targeted metabolomics platform and self‐built database, we compared and found differences in serum metabolic profiles between different groups. Endogenous metabolites in serum were analyzed qualitatively and quantitatively in Multiple Reaction Monitoring (MRM) mode using a triple quadrupole mass spectrometer.

For the gut microbiome, 16S rRNA diversity sequencing was used in our study. Briefly, the genomic DNA was extracted by using the cetyltrimethylammonium bromide (CTAB) method (Yu et al., 2017), and then DNA purity was determined by agarose gel electrophoresis. Thereafter, PCR amplification was performed using Phusion® High‐Fidelity PCR Master Mix with GC Buffer (New England Biolabs) and genomic DNA. Finally, the 16S rRNA diversity sequencing was conducted on the NovaSeq6000 platform.

### 2.3 Statistics analysis

For the targeted metabolomic analysis, the principal component analysis (PCA) model was initially used to observe the overall metabolic differences and the degree of variability among different groups, including quality control samples. Further, the orthogonal partial least‐squares discrimination analysis (OPLS‐DA), a multivariate statistical analysis method for supervised pattern recognition, was employed to compare metabolic profiles in the various groups. Differentially expressed metabolites were screened using a criterion of VIP >1, Log2FC (fold change) >1 and *p* < .05. Based on these differentially expressed metabolites, an enriched pathway analysis using the KEGG database was performed, in which statistical significance was determined by the p‐value of the hypergeometric test. For the 16S rRNA diversity sequencing, alpha diversity was calculated using Qiime software (Version 1.9.1). The species with significant differences between groups were tested and mapped using R software. The Spearman correlation analysis between host serum metabolites and microbiota was calculated using the cor function of the R software.

## 3 RESULTS

### 3.1 The protective effects of naringenin against NASH

**FIGURE 1:**
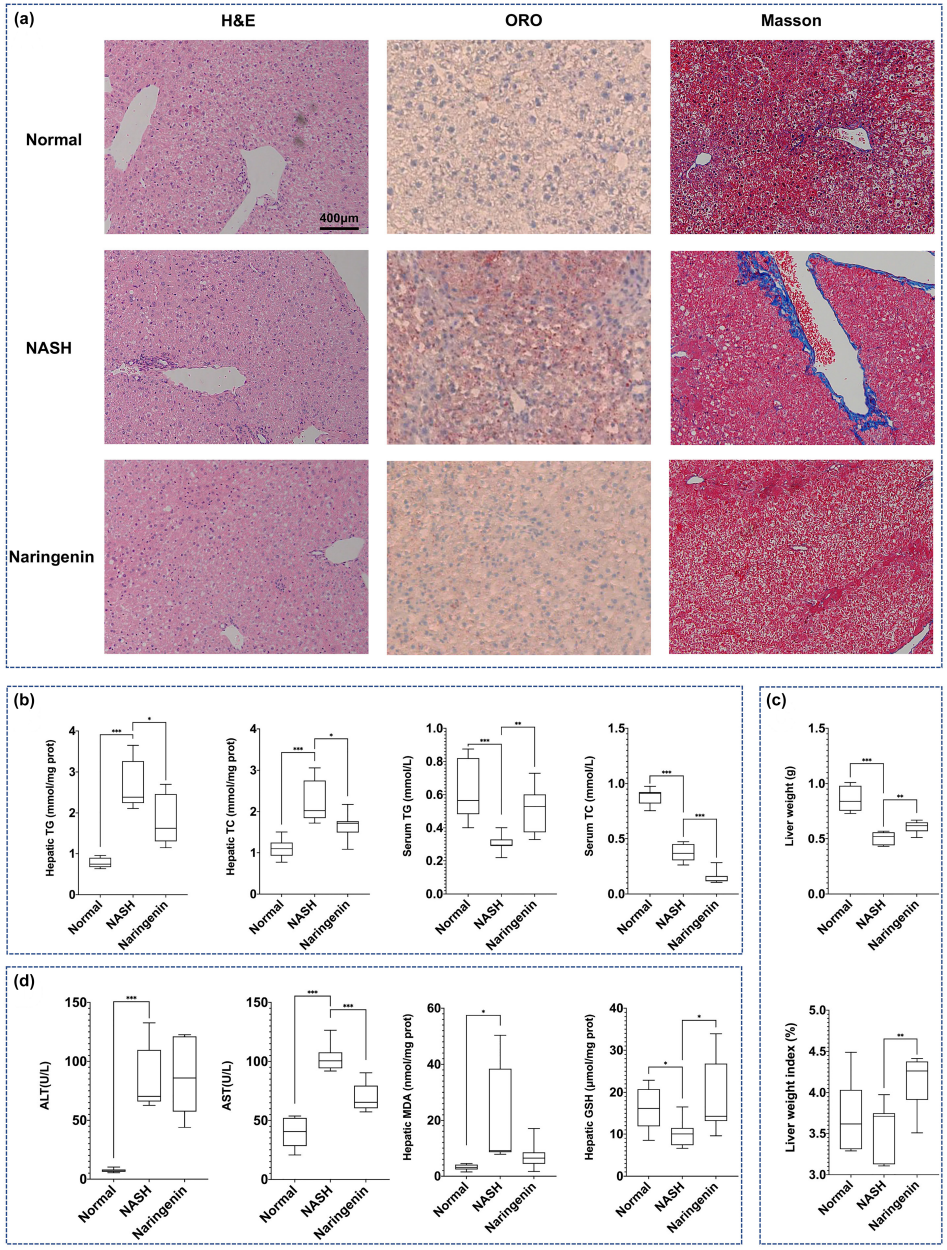
Naringenin ameliorates many aspects of NASH. (a) Pathological alterations of livers in different groups (ORO, H&E, and Masson staining; scale bar represents 200 μm length); Biochemical indicators of liver function, including (b) hepatic TG/TC contents and serum TG/TC levels; (c) liver weight and liver weight/body weight index; and (d) serum ALT/AST levels and hepatic MDA and GSH contents. (Values are expressed as box‐and‐whisker plots: ***p < .001, **p < .01, *p < .05).

As displayed in Figure 1a, compared with the Normal group, mice in the NASH group had more fat accumulation (Oil Red O staining, ORO), neutrophil infiltration (H&E staining), and blue collagen fibers (Masson staining) in the liver, whereas these histopathological alterations were prevented in mice fed with an MCD diet supplemented with naringenin.

Figure 1b–d displays biochemical indicators of liver function in the different groups. Consistent with the results of ORO staining, the contents of TG and TC in the liver of the NASH group were significantly higher than those of the Normal group, which was significantly recovered with naringenin intervention (Figure 1b). We observed that naringenin intervention restored serum TG levels but further decreased serum TC levels compared to the NASH group (Figure 1b).

The MCD diet resulted in a significant decrease in liver weight; however, naringenin supplementation significantly prevented this change (Figure 1c). This trend was even more pronounced when the liver weight was normalized to body weight (Figure 1c).

As shown in Figure 1d, compared with the Normal group, the levels of ALT and AST in the NASH group were dramatically increased, suggesting abnormal liver function in the NAFLD mice. In addition, the contents of hepatic MDA and GSH, which reflect oxidative stress and antioxidant capacity, respectively, were significantly abnormal in MCD diet mice. Supplementation with naringenin significantly rescued AST and GSH levels, with a trend of recovering MDA levels (Figure 1d).

### 3.2 Analysis of host serum metabolomics

A total of more than 700 metabolites were detected in this study using amide‐negative mode, amide‐positive mode, T3‐negative mode, and T3‐positive mode (Figure S1). A quality control (QC) sample, which was prepared by mixing the sample extracts and embedded in every tenth sample, was used to monitor the repetitiveness of the instrument. The results showed high consistency of the QC total ion chromatographs (TIC) (Figure S1), indicating the stability of the signal during the analysis process.

**FIGURE 2:**
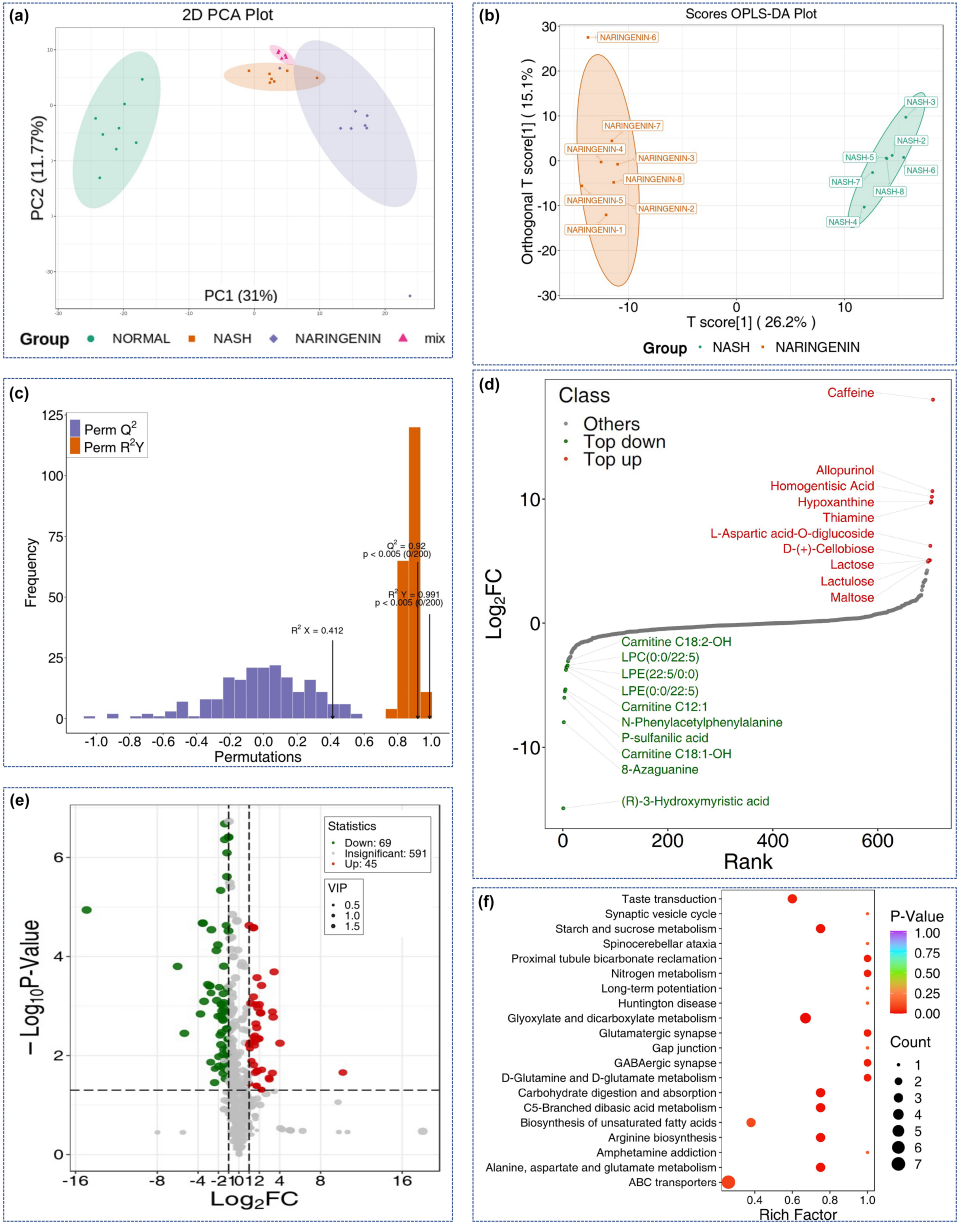
Analysis of host serum metabolic profiles in mice in different groups. (a) Overall PCA map; (b) OPLAS‐DA scoring map between NASH and Naringenin groups; (c) Model validation of OPLAS‐DA scoring map between NASH and Naringenin groups; (d) The top differentially expressed metabolites with the largest FC values between NASH and Naringenin groups; (e) Volcano map of differentially expressed metabolites between NASH and Naringenin groups; (f) Enriched pathway analysis using the KEGG database.

As shown in Figure 2a,b samples from the Normal group, NASH group, and Naringenin group were completely separated on PCA score and OPLS‐DA graphs, highlighting the significant effects of MCD diet feeding and naringenin intervention on the serum metabolic profiles of the mice. In the OPLS‐DA model, *R* ^2^ *Y* and *Q* ^2^ values, which represent accuracy and efficiency, were 0.991 (*p* < .005) and 0.92 (*p* < .005), respectively (Figure 2c), indicating the model had robust discriminative ability. Figure 2d displays the differential metabolites with the largest values of FC between the NASH and Naringenin groups. The upregulated metabolites in the Naringenin group, compared to the NASH group, with the top 10 FC values were caffeine, homogentisic acid, L‐aspartic acid‐O‐diglucoside, lactulose, lactose, D‐(+)‐cellobiose, maltose, D‐trehalose, isopentenyladenine‐7‐N‐glucoside, and D‐(+)‐sucrose. The top 10 downregulated metabolites, in terms of FC, were (R)‐3‐hydroxymyristic acid, carnitine C18:1‐OH, N‐phenylacetylphenylalanine, carnitine C12:1, LPE (0:0/22:5), LPE (22:5/0:0), LPC (0:0/22:5), carnitine C18:2‐OH, 2‐hydroxyphenylacetic acid, and 4‐hydroxy‐3‐methylbenzoic acid. In addition, a volcano plot was constructed to exhibit the statistical significance and degree of difference in the metabolic expression levels between the NASH group and the Naringenin group by using combined screening criteria of fold change (FC) ≥2 or ≤0.5 projection of variable importance (VIP) of OPLS‐DA model > 1, and *p* < .05. As shown in Figure 2e, among the detected metabolites in the Naringenin group compared to the NASH group, 591 were not significantly altered, 69 were downregulated, and 45 were upregulated. As shown in Figure 2f, the relevant major pathways could be classified into proximal tubule bicarbonate reclamation, nitrogen metabolism, glutamatergic synapse, GABAergic synapse, and D‐glutamine and D‐glutamate metabolism.

Numerous differentially expressed metabolites were screened in the Normal/NASH and NASH/Naringenin comparisons based on the criteria of VIP >1, *p* < .001, and fold change >2. Of these metabolites, a total of 37 were present in both comparisons (Table 1). Our results clearly showed that the levels of 3‐ureidopropionic acid, PC (16:1e/8,9‐EpETE), KN‐93, pinolenic acid, EPA, FFA (18:3), FFA (18:4), MG (0:0/20:5/0:0), LPE (0:0/18:3), carnitine C8:1, and carnitine C9:1‐OH were significantly decreased in the NASH group compared with the Normal group, and that naringenin intervention helped restore the levels toward those of the Normal group. In contrast, the NASH group had significantly higher levels of 1‐aminocyclohexanoic acid, LPE (0:0/22:5), 2‐hydroxycaprylic acid, 2‐hydroxyphenylacetic acid, and FAHFA (8:0/10:0) than the Normal group, and the altered levels of these metabolites were restored by naringenin supplementation. Interestingly, the contents of a series of carnitine family compounds were substantially decreased in the NASH group when compared with the Normal group, and these levels were further reduced with naringenin treatment. In addition, the levels of some metabolites were dramatically elevated in the NASH group compared with the Normal group and further increased by naringenin intervention.

**TABLE 1:** Differentially expressed metabolites shared between Normal/NASH and NASH/Naringenin comparisons.

	Normal vs. NASH				NASH vs. Naringenin			
Compounds	VIP	*p*‐Value	FC	Type	VIP	*p*‐Value	FC	Type
3‐Ureidopropionic acid	1.299	.00002	0.32	Down	1.007	.00422	2.64	Up
PC (16:1e/8,9‐EpETE)	1.355	.00005	0.07	Down	1.598	.00020	10.91	Up
KN‐93	1.243	.00348	0.06	Down	1.709	.00563	16.25	Up
Pinolenic acid	1.524	.00015	0.27	Down	1.536	.00455	3.07	Up
EPA	1.458	.00034	0.16	Down	1.600	.00027	3.42	Up
FFA (18:3)	1.493	.00056	0.24	Down	1.603	.00455	3.69	Up
FFA (18:4)	1.541	.00240	0.09	Down	1.747	.00109	3.66	Up
MG (0:0/20:5/0:0)	1.248	.00141	0.26	Down	1.652	.00138	4.37	Up
LPE (0:0/18:3)	1.359	.00037	0.49	Down	1.748	.00003	2.67	Up
Carnitine C8:1	1.595	.00001	0.09	Down	1.551	.00529	2.89	Up
Carnitine C9:1‐OH	1.595	.00002	0.09	Down	1.620	.00457	3.89	Up
1‐Aminocyclohexanoic acid	1.617	.00000	4.82	Up	1.619	.00000	0.29	Down
LPE (0:0/22:5)	1.390	.00006	2.57	Up	1.892	.00002	0.09	Down
2‐Hydroxycaprylic acid	1.393	.00597	2.46	Up	1.603	.00784	0.45	Down
2‐Hydroxyphenylacetic acid	1.302	.00103	3.20	Up	1.802	.00039	0.14	Down
FAHFA (8:0/10:0)	1.501	.00003	2.27	Up	1.469	.00002	0.42	Down
Carnitine C12:0	1.420	.00004	0.24	Down	1.373	.00055	0.15	Down
Carnitine C6:0	1.487	.00001	0.29	Down	1.753	.00181	0.32	Down
Carnitine C18:2‐OH	1.357	.00014	0.41	Down	1.533	.00037	0.12	Down
Carnitine C18:1	1.478	.00001	0.36	Down	1.670	.00112	0.36	Down
Carnitine C18:2	1.338	.00011	0.49	Down	1.667	.00194	0.34	Down
Carnitine C16:1	1.499	.00006	0.24	Down	1.707	.00157	0.30	Down
Carnitine C16:2	1.287	.00082	0.38	Down	1.552	.00750	0.36	Down
Carnitine C14:0	1.581	.00001	0.22	Down	1.737	.00016	0.36	Down
Carnitine C14:1	1.445	.00001	0.28	Down	1.684	.00386	0.28	Down
Carnitine C12:1	1.253	.00343	0.32	Down	1.739	.00145	0.07	Down
Carnitine C10:1	1.361	.00006	0.42	Down	1.611	.00183	0.33	Down
Carnitine C14:2:DC	1.492	.00004	0.23	Down	1.700	.00343	0.31	Down
Carnitine C18:1‐OH	1.547	.00000	0.23	Down	1.779	.00016	0.02	Down
Carnitine C18‐OH	1.394	.00001	0.37	Down	1.620	.00949	0.34	Down
4‐Guanidinobutyric Acid	1.622	.00220	24,691	Up	1.706	.00167	10.24	Up
N‐Acetyl‐L‐Glutamic Acid	1.424	.00104	10.59	Up	1.487	.00065	2.70	Up
6‐Hydroxynicotinic Acid	1.630	.00157	3993	Up	1.471	.00093	4.01	Up
L‐2‐amino‐6‐oximelic acid	1.236	.00436	2.70	Up	1.407	.00228	3.15	Up
2‐pyrimidine methanol	1.322	.00113	13.07	Up	1.296	.00613	2.01	Up
N‐Phenylacetylphenylalanine	1.471	.00359	34.58	Up	1.772	.00356	0.02	Up
Docosahexaenoic Acid Glycine	1.273	.00144	6.02	Up	1.505	.00094	3.15	Up

Abbreviation: FC, fold change.

### 3.3 Analysis of the composition and richness of the gut microbiome

**FIGURE 3:**
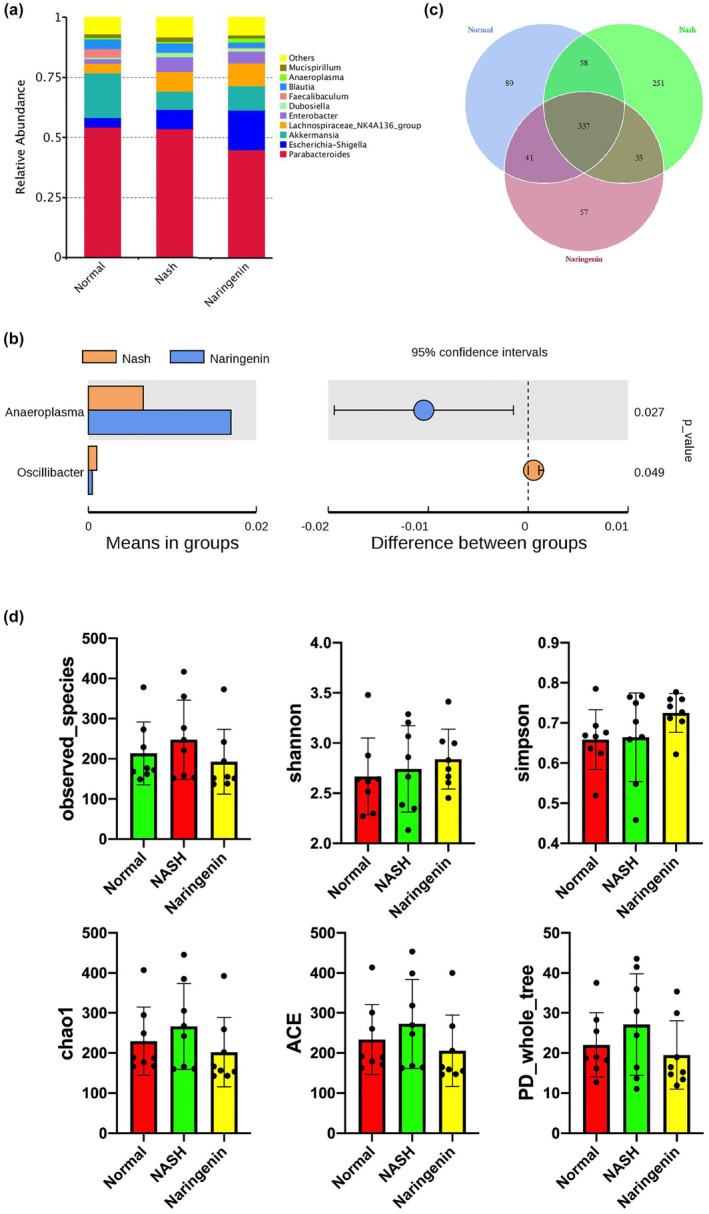
Analysis of the composition and richness of the gut microbiome in different groups (a) Histogram of relative abundance of species at the genus level; (b) Differential gut microbiome richness between the NASH group and the Naringenin group at the genus level; (c) Venn diagram; (d) Alpha Diversity Index (observed species, Shannon index, Simpson index, Chao1 index, ACE index, and PD whole tree index).

The intestinal contents of each mouse were analyzed using 16S rDNA amplicon sequencing. The relative abundance of the top ten most abundant microbial populations, described at the genus level, is displayed in Figure 3a. The relative abundance of *Enterobacter*, *Dubosiella*, and *Mucispirillum* was increased in the NASH group compared with the Normal group, which could be reversed by naringenin treatment. The composition of *Parabacteroides*, *Blautia*, and *Anaeroplasma* was not altered between the NASH group and the Normal group but was decreased by naringenin intervention. The relative abundance of *Escherichia–Shigella* and *Lachnospiraceae_NK4A136_group* was elevated in NASH group compared with Normal group and was further increased in mice treated with naringenin. In addition, *Faecalibaculum* was not altered by naringenin treatment. The alteration of the genus of *Anaeroplasma* and *Oscillibacter* reached statistical differences between the NASH group and the Naringenin group (*p* < .05; Figure 3b). The number of detected operational taxonomic units (OTUs) was displayed using a Venn diagram, showing a total of 337 identical OTUs across all groups (Figure 3c). Alpha diversity analysis showed that observed species, chao1, ACE, and PD whole tree indexes were elevated in the NASH group in comparison with Normal group, which was restored by naringenin intervention (Figure 3d).

### 3.4 Correlation analysis of gut microbiota and host serum metabolomics

**FIGURE 4:**
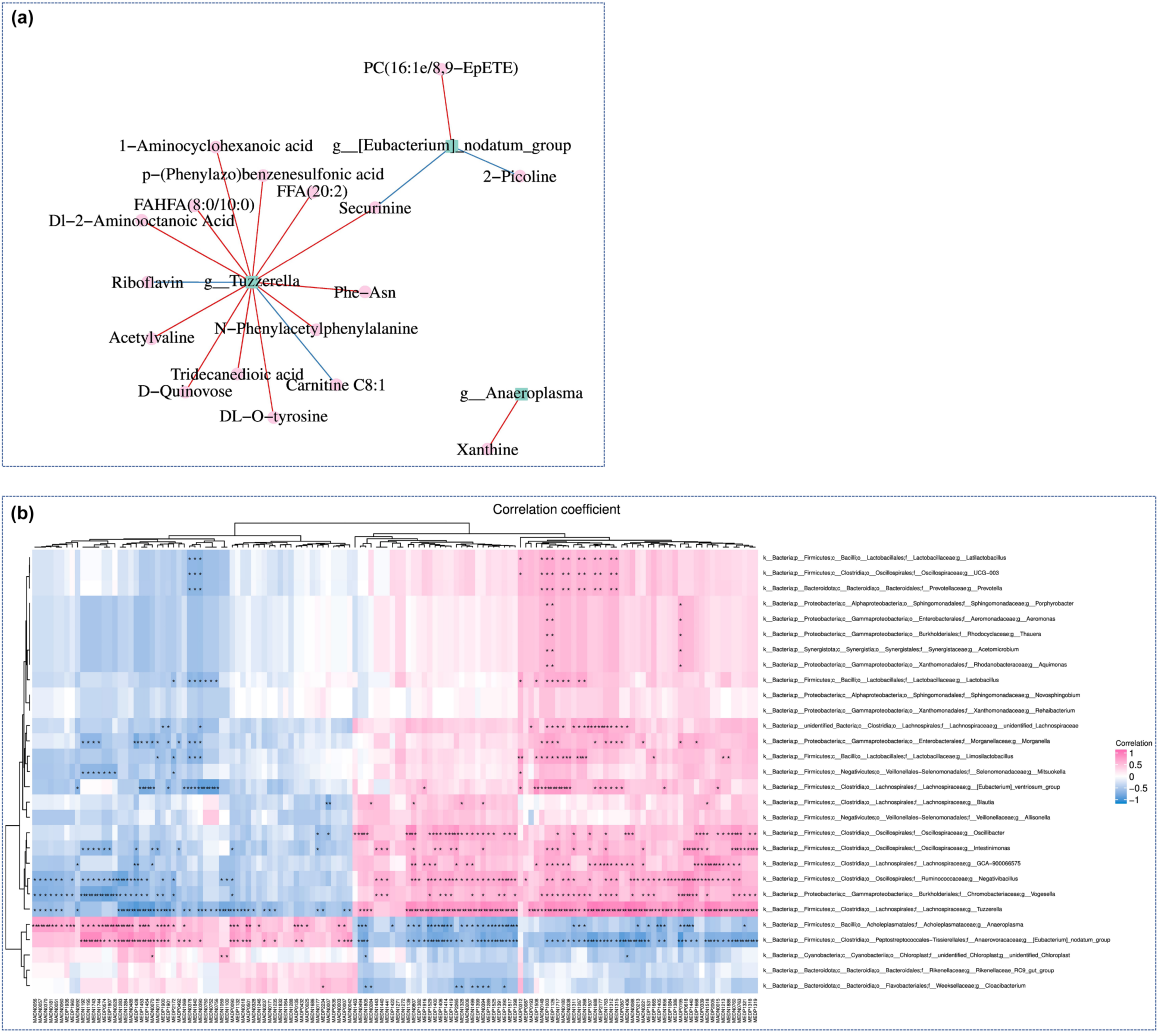
Correlation analysis of gut microbiota (genus levels) and host serum metabolites between the NASH group and the Naringenin group (a) Correlation network diagram (|*r*| > =0.8 and p‐value<0.05; metabolites are shown in pink and microbes in light green; red connecting lines indicate a positive correlation and blue lines indicate a negative correlation, with thicker lines indicating a greater correlation); (b) Spearman correlation cluster heatmap of the differential gut microbiome and differential metabolites at the genus level. The central heatmap shows the magnitude of the Spearman correlation between differential microbes and differential metabolites. *p < .05, **p < .01. The abscissa represents metabolites, and the ordinate represents microbes.

The potential relationship between the gut microbiota changes at the genus level and the metabolites in the serum was analyzed. As shown in Figure 4a, a network diagram was used to show correlations based on a criterion of |*r*| > =0.8 and *p*‐value < .05. Metabolites are shown in pink, while microbes are in light green. Red connecting lines indicate a positive correlation, and blue lines indicate a negative correlation, with line thickness being indicative of correlation strength. *Anaeroplasma* was positively related with xanthine, and the *[Eubacterium] nodatum group* was positively related with PC (16:1e/8,9‐EpETE) but negatively related with 2‐picoline and securinine. *Tuzzerella* was closely related to a number of metabolites, including 12 positively correlated compounds (N‐phenylacetylphenylalanine, 1‐aminocyclohexanoic acid, Phe‐Asn, DL‐O‐tyrosine, FAHFA (8:0/10:0), acetylvaline, tridecanedioic acid, FFA (20:2), p‐(phenylazo)benzenesulfonic acid, securinine, D‐quinovose, Dl‐2‐aminooctanoic acid) and 2 negatively correlated compounds (riboflavin and carnitine C8:1). In addition, Spearman correlation hierarchical cluster analysis was also employed to comprehensively assess the coefficient of association between differential microbiota and differential metabolites. The results were plotted and displayed using a heatmap (R software's ComplexHeatmap package). As displayed in Figure 4b, there exist abundant correlations between differential microbiota and metabolites (*p < .05, **p < .01). It is noteworthy that genus levels of *Anaeroplasma*, *[Eubacterium] nodatum group*, and *Tuzzerella* were the three bacteria genera that most influenced the host serum metabolome.

**FIGURE 5:**
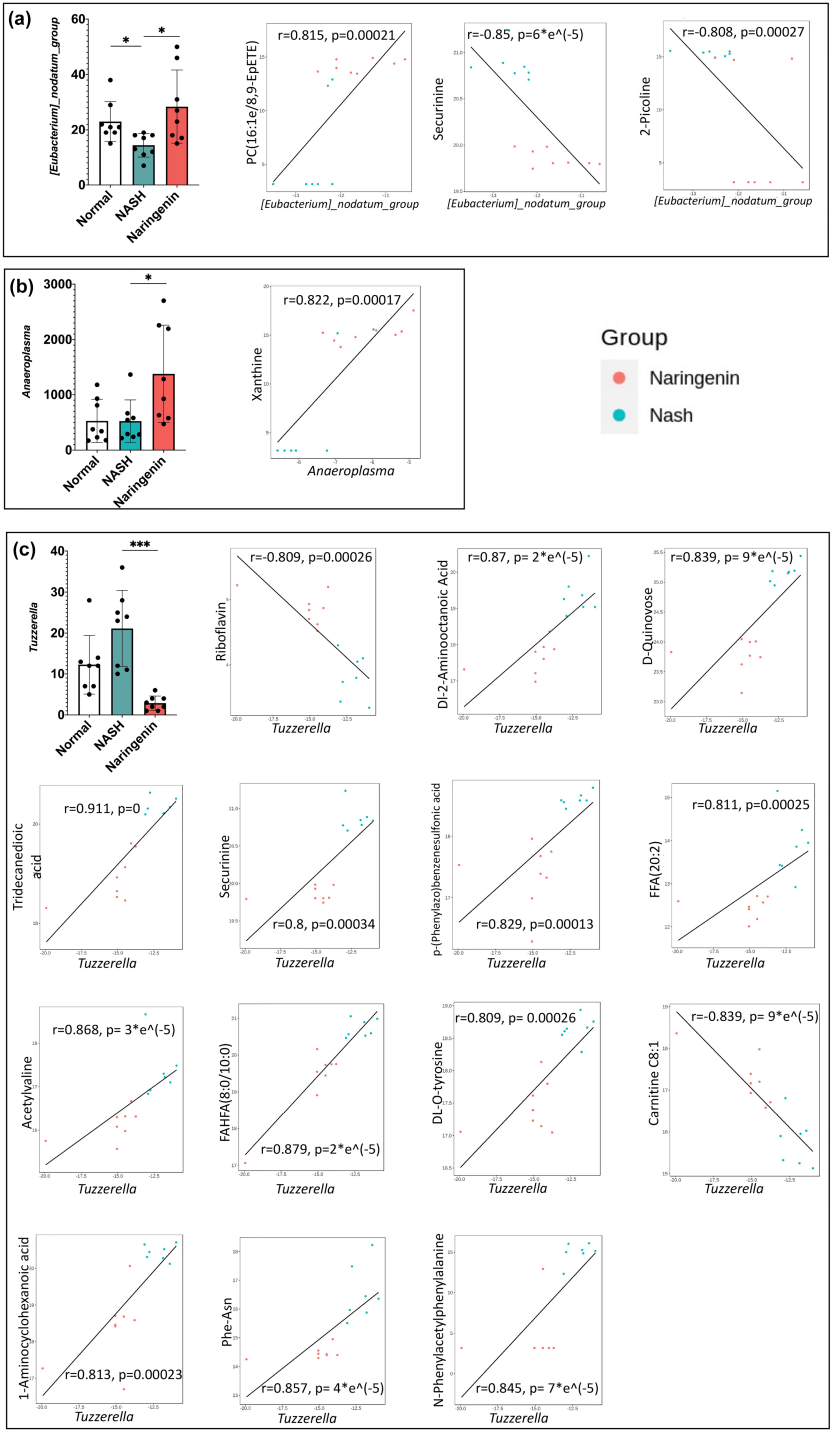
The most significantly correlated gut microbes and host serum metabolites.

Scatter diagrams were constructed to display the most associated gut microbes and host serum metabolites (Figure 5). The levels of the gut *[Eubacterium] nodatum group* and *Tuzzerella* were altered in the NASH group compared with the Normal group, and these were restored by naringenin intervention. *Anaeroplasma* content was not changed between the Normal group and the NASH group but was significantly increased in the Naringenin group compared with the NASH group. Consistent with the results of the correlation network diagram and Spearman correlation cluster heatmap (Figure 4), the three genera of microbiota were closely related to a series of serum metabolites.

## 4 DISCUSSION

There is a significant unmet clinical need to create bioactive compounds to prevent NASH, as the FDA does not have an approved drug therapy for this disease. In our study, we found that naringenin, a natural flavanone with broad pharmacological activities, could effectively suppress the progression of NASH, ameliorating cell injury, lipid deposition, and oxidative stress in the liver. It has been reported that the MCD diet promotes hepatic steatosis by increasing hepatic fatty acid uptake and decreasing lipoprotein secretion, resulting in lower blood TC/TG and severe intrahepatic lipid accumulation in MCD‐fed mice (Rinella et al., 2008). Indeed, our results showed that hepatic TG and TC in the NASH group were higher than in the Normal group. Their liver weight and serum TC/TG levels were decreased in the NASH group compared with the Normal group, which is consistent with previous studies (Xiong et al., 2021). Here we show that naringenin intervention can exert a beneficial protective effect and reverse these changes.

Furthermore, we use omics methods to help elucidate potential mechanisms behind the beneficial effects of naringin. To our knowledge, there are no multi‐omics reports on the systematic protective effects of naringenin. In our study, to avoid any misdirection, we used a local database built from thousands of standard compounds, which has the advantage of high throughput and accuracy. Herein, we detected more than 700 metabolites, and PCA modeling showed that the metabolic profiles were significantly separated among the three groups. Among the differentially expressed metabolites, it is noteworthy that KN‐93 was increased by 16.25 fold in the Naringenin group. KN‐93 is a selective Ca^2+^/calmodulin‐dependent kinase II (CaMKII) inhibitor that permeates cells and inhibits the phosphorylation activity of CaMKII. CaMKII belongs to a multifunctional serine/threonine protein kinase and has a wide range of functions. KN‐93 has been well studied in heart diseases and has been shown to have a cardioprotective effect. In our study, it was interesting to find that KN‐93 was barely detected in the blood of MDC‐fed mice (all of them were below the lower limit of quantitation except for one mouse). The levels of KN‐93 were dramatically decreased by MCD feeding but were reversed with naringenin intervention. It was reported that KN‐93 can inhibit the proliferation of human hepatic stellate cells (An et al., 2007), a subpopulation cell in the liver that plays a critical role in dictating NAFLD outcomes (Wiering et al., 2023). This implies that naringin may alleviate NASH by upregulating KN‐93 levels and suppressing hepatic stellate cell proliferation. Conversely, among the compounds downregulated by naringenin, LPE (0:0/22:5) was the metabolite with the most significant change. LPE is a hydrolyzed product of PE by phospholipase A2 and a neurotrophic activator mediated by mitogen‐activated protein kinase (MAPK) signaling (Nishina et al., 2006). Yamamoto et al. found that an LPE is involved in the inhibition of lipolysis and fatty acid biosynthesis and may play a pathological role in the progression of NAFLD (Yamamoto et al., 2022). Thus, we speculate that naringenin may decrease the level of LPE and hamper the development of fatty liver and hepatic inflammation. A comprehensive lipidomic study of LPE is required to explore the effects of naringenin on lipid metabolism.

*Parabacteroides distasonis* is a beneficial bacterium that can ameliorate hepatic fibrosis and metabolic dysfunctions by modulating bile acid metabolism (Zhao et al., 2023). *Escherichia/Shigella*, however, is a Gram‐negative inflammation‐inducible genus that is elevated in NAFLD (Yin et al., 2013). In our results, it was unexpected to find that naringenin treatment decreased the abundance of beneficial *Parabacteroides* but increased the abundance of harmful *Escherichia/Shigella*, making it difficult to interpret the beneficial effects of naringenin. In contrast, the abundance of *Akkermansia*, a beneficial microorganism with diverse functions (Depommier et al., 2019), and *Enterobacter*, which aggravates metabolic disease by inducing lipotoxicity and inflammation (Jin et al., 2022), was abnormal in NASH mice but was reversed to beneficial levels in mice with naringenin, suggesting a potential protective mechanism of naringenin.

With the accumulation of studies on the gut‐liver axis, it is well established that gut microbiota can interact with the host metabolism and thus impact the physiology and thus produce pathological states in the liver. A study in mice found that the protective effect and potential mechanisms of Astragali Radix against cisplatin‐induced liver injury were related to modulation of the host microbiome and gut metabolome (Wang et al., 2022). In our current study, we found that 3 types of microbiota genera were closely associated with 18 different metabolites.

Our results showed that the level of *[Eubacterium] nodatum group*, was reduced in the NASH group which manifested severe hepatic inflammation when compared with the Normal group. Naringenin could completely reverse this trend, suggesting its protective role in alleviating NASH might be related to the regulation of the *[Eubacterium] nodatum group*. Yan et al. reported that there was a significant negative correlation between the relative abundance of the *[Eubacterium] nodatum group* and proinflammatory cytokines (IL‐6 and TNF‐α) in LPS‐induced systemic inflammation, implying that this bacteria genus may exert a resistant effect on inflammation (Wei et al., 2022). This evidence suggests that the anti‐inflammatory action of naringenin may be related to regulating *[Eubacterium] nodatum group* abundance. In Spearman correlation analysis, this genus was positively associated with PC (16:1e/8,9‐EpETE), which belongs to the category of phosphatidylcholine; however, the specific biological function of this lipid has not been explored to our knowledge. The close relation between *[Eubacterium] nodatum group* and PC (16:1e/8,9‐EpETE) suggests that naringenin may enhance the amount of *[Eubacterium] nodatum group*, which then results in an increased level of PC (16:1e/8,9‐EpETE) in the peripheral circulation of the host.

Meanwhile, the *[Eubacterium] nodatum group* was negatively associated with 2‐picoline and securinine. The pharmacological effect of 2‐picoline has not been reported, whereas securinine, an alkaloid derived from the leaf of *Securinega suffruticosa* Rehd., has been extensively studied and is demonstrated to have neuroprotective effects (Neganova et al., 2016), enhance macrophage clearance as a GABA_A_ receptor antagonist (Lubick et al., 2007), and induce apoptosis in HeLa cells (Stefanowicz‐Hajduk et al., 2016). In clinical practice, securinine is frequently used in treating polio sequelae and facial nerve palsy, but it also has adverse effects such as liver damage (L. Yu et al., 2021). Our study found that the level of securinine increased in the NASH group. This was not suppressed by naringenin, implying that securinine may play a deleterious role in the progression of NASH. Naringenin may ameliorate the disease by decreasing the circulating content of securinine, which is closely related to the regulation of the *[Eubacterium] nodatum group*. However, the effects of these endogenous compounds on NASH and their interaction with the *[Eubacterium] nodatum group* remain unknown and might be an interesting area for future investigation.

Our results also showed that the content of intestinal *anaeroplasma* was positively related to the host's serum level of xanthine. Xanthine levels are significantly increased in advanced NASH compared to the early stages (Ioannou et al., 2020), which is in accordance with our finding. Yusuke et al. reported that the enzyme activity of xanthine oxidoreductase (XOR) is induced in the plasma of NASH patients, accelerating the catalytic reactions of hypoxanthine to xanthine and xanthine to uric acid (Kawachi et al., 2021). An XOR inhibitor was demonstrated to suppress the development of NASH (Nakatsu et al., 2015). Herein, we suppose that naringenin may increase the serum level of xanthine by regulating the enzyme activity of XOR. Previous studies showed that *Anaeroplasma* decreased significantly in high‐fat‐diet‐induced NAFLD rodent models (Velazquez et al., 2019), suggesting a beneficial effect of this genus. Herein, the MCD diet induced NASH, a severe stage of NAFLD, but did not alter the composition of the *Anaeroplasma* genus. However, naringenin significantly increased the level of *Anaeroplasma*, and showed a strong correlation between xanthine and *Anaeroplasma*, suggesting that *Anaeroplasma* may influence the physiological change of host serum xanthine. Investigating the interaction of *Anaeroplasma* and xanthine in the treatment of naringenin is another interesting area for further study.

In the present study, *Tuzzerella* alterations showed the highest number of associations with other host endogenous metabolites. A recent publication also used the MCD diet to establish the NASH model, and they found that *Poria cocos* polysaccharides could significantly decrease the abundance of *Tuzzerella* genus and improve NASH (Tan et al., 2022), which is consistent with our findings. The biological function of *Tuzzerella* remains unknown, including how it may help in the treatment of NASH. In a multi‐omics study of colorectal cancer, *Tuzzerel* was also the microbiol genus that was most associated with other genes and metabolites, including a negative association with serum uric acid levels and the expression of BEST4 and DGKB genes (Lo et al., 2023). In our correlation analysis, we found a high correlation between up to 14 species of host serum metabolites and intestinal abundance of the *Tuzzerella* genus. Among the 14 types of endogenous metabolites, FAHFA (8:0/10:0), FFA (20:2), Carnitine C8:1 and Tridecanedioic acid belong to the category of fatty acyl; securinine, acetylvaline, DL‐O‐tyrosine, and Phe‐Asn belong to the category of amino acid derivatives; other metabolites associated with *Tuzzerella* are N‐Phenylacetylphenylalanine, 1‐Aminocyclohexanoic acid, p‐(Phenylazo)benzenesulfonic acid, D‐Quinovose, Dl‐2‐Aminooctanoic Acid, and Riboflavin. These results demonstrate that *Tuzzerella* may play an important role in influencing host metabolism.

It is noteworthy that securinine has correlations with both the *Tuzzerella* and *[Eubacterium] nodatum groups*; however, the correlation is opposite. Securinine is an alkaloid derived from *Phyllanthus amarus* that has been reported to exert antifungal activity (Singh et al., 2008), therapeutic activity for acute myeloid leukemia (Gupta et al., 2011), and anticancer activities (Liu et al., 2022). To our knowledge, several interesting questions, such as the role of securinine in the development of NASH, the interplay between securinine and the microbiota, and the regulatory role of naringenin on securinine, have not been reported yet and deserve further exploration.

Overall, the identified genus‐metabolite crosslinks in our study provide novel potential mechanistic insights into the protective effects of naringenin against NASH. However, the beneficial effects of naringenin on NASH remain to be further verified in more rodent models since MCD‐induced NASH does not fully match the characteristics of human NASH. Secondly, the use of antibiotics in mouse models will help verify whether naringenin‐induced alterations of serum metabolites are caused by specific gut bacteria and whether naringenin‐induced crosstalk between serum metabolites and gut bacteria is required for the beneficial effects of naringenin on NASH. Lastly, the mechanisms by which naringenin regulates the expression of endogenous metabolites are not clear, and more specific studies are required.

## 5 CONCLUSIONS

In summary, this study provides mechanistic insights into how naringenin suppresses NASH development. We demonstrated that naringenin intervention substantially restored the serum metabolome and gut microbiome in a preclinical NASH setting. Through the integration of multi‐omics data, we found that the genera levels of *Anaeroplasma*, *[Eubacterium] nodatum group*, and *Tuzzerella* were significantly altered by naringenin treatment and closely correlated with 17 endogenous metabolites in the host's serum, such as fatty acyl and amino acid derivatives. Our findings provide information that may aid in the further development of naringenin treatments; however, further studies on the crosstalk between host and gut microbiota are warranted.

## AUTHOR CONTRIBUTIONS

**Peng Cao:** Conceptualization (equal); data curation (equal); formal analysis (equal); funding acquisition (equal); investigation (equal); methodology (equal); writing – original draft (equal). **Ming Yue:** Conceptualization (equal); resources (equal). **Yuanlei Cheng:** Conceptualization (equal); formal analysis (equal). **Mitchell A. Sullivan:** Investigation (equal); writing – review and editing (equal). **Wen Chen:** Investigation (equal). **Huifan Yu:** Investigation (equal). **Fei Li:** Investigation (equal). **Sanlan Wu:** Investigation (equal). **Yongning Lv:** Investigation (equal). **Xuejia Zhai:** Conceptualization (equal); supervision (equal). **Yu Zhang:** Conceptualization (equal); supervision (equal).

## FUNDING INFORMATION

This study was supported by National Natural Science Foundation of China (Grant No. 81903901 to Dr. Peng Cao), the Hubei Provincial Natural Science Foundation (Grant No. 2023AFB800 to Dr. Peng Cao), the Dawning Program of Wuhan Knowledge Innovation Special Project (Grant No. 2022020801020467 to Dr. Peng Cao), the Open Project of the Hubei Key Laboratory of Wudang Local Chinese Medicine Research (Grant No. WDCM2023003 to Dr. Peng Cao), and the Open Project Funding of Hubei Key Laboratory of Biological Targeted Therapy (Grant No. 2022swbx015 to Dr. Peng Cao). Dr. Mitchell Sullivan is supported by an Advance Queensland Industry Research Fellowship and the Mater Foundation (Equity Trustees and the L. G. McCallam Est and George Weaber Trusts).

## CONFLICT OF INTEREST STATEMENT

The authors declare no conflicts of interest.

## ETHICS STATEMENT

The animal study was reviewed and approved by the Institutional Animal Care and Use Committee of Tongji Medical College, Huazhong University of Science and Technology.

## Supporting information


Figure S1.

## Data Availability

Data are available by contacting the corresponding author (Dr. Peng Cao).
