# Supplementary material for: Naringenin prevents non‐alcoholic steatohepatitis by modulating the host metabolome and intestinal microbiome in MCD diet‐fed mice
Source: Food Sci Nutr. 2023 Sep 27;11(12):7826–40. doi: 10.1002/fsn3.3700 (PMC10724642; doi:10.1002/fsn3.3700)
Supplement: Supplementary file 1 — Figure S1. [file FSN3-11-7826-s001.docx]

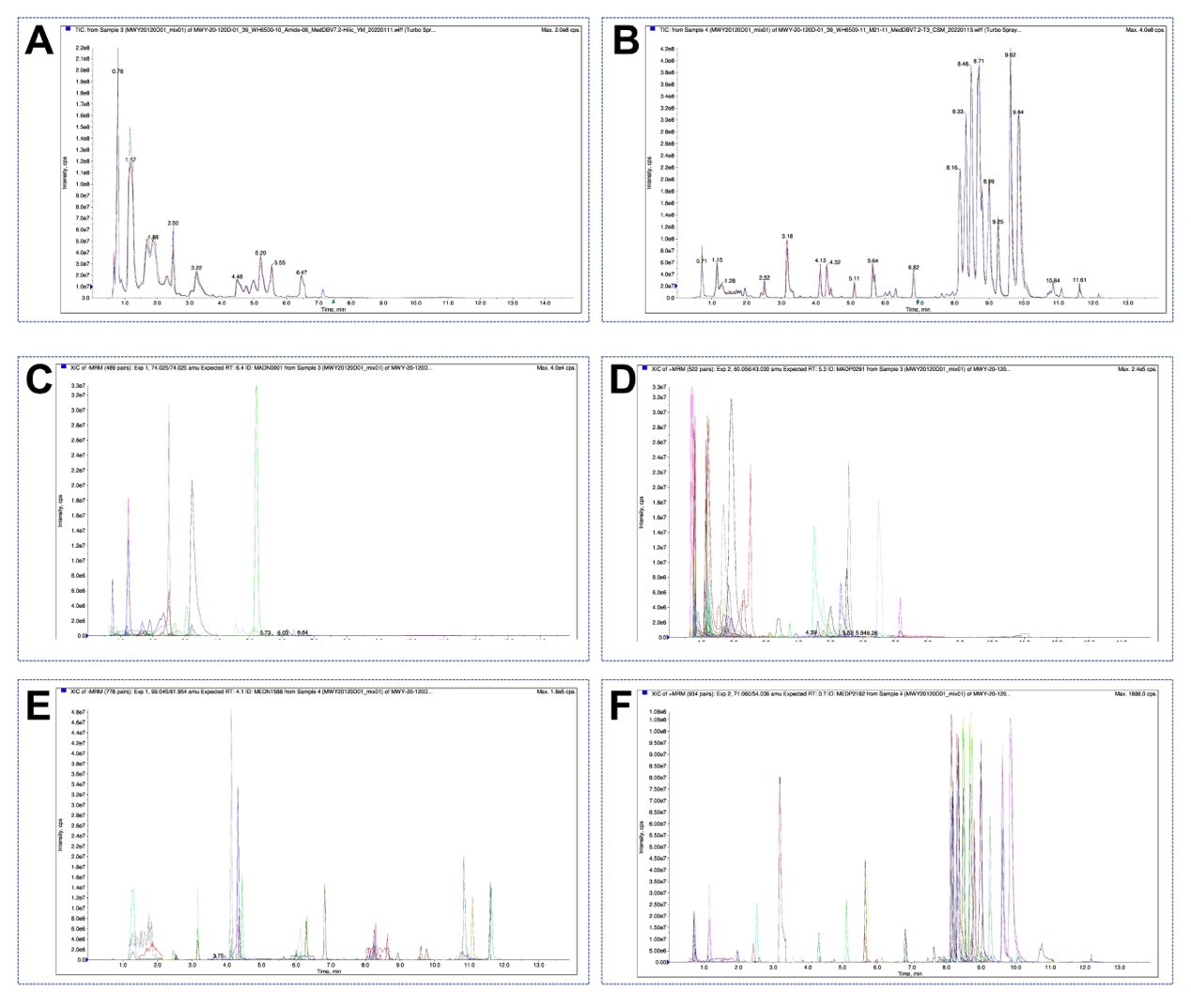


Supplemental figure 1. The chromatograms of targeted metabolomics in (A) positive and (B) negative modes, respectively. The multi-peak maps in MRM detection using (C) Amide-negative mode, (D) Amide-positive mode, (E) T3-negative mode and (F) T3-positive mode.
